# Impact of wait times on late postprocedural mortality after successful transcatheter aortic valve replacement

**DOI:** 10.1038/s41598-022-09995-z

**Published:** 2022-04-08

**Authors:** Vincent Roule, Idir Rebouh, Adrien Lemaitre, Rémi Sabatier, Katrien Blanchart, Clément Briet, Mathieu Bignon, Farzin Beygui

**Affiliations:** 1 CHU de Caen Normandie, Service de Cardiologie, 14000 Caen, France; 2 INSERM UMRS 1237, GIP Cyceron, 14000 Caen, France; 3 ACTION Study Group, Cardiology Department, Pitié-Salpêtrière University Hospital, Paris, France; 4 Cardiology Department, Caen University Hospital, Avenue Cote de Nacre, 14033 Caen, France

**Keywords:** Interventional cardiology, Disease-free survival

## Abstract

Wait times are associated with mortality on waiting list for transcatheter aortic valve replacement (TAVR). Whether longer wait times are associated with long term mortality after successful TAVR remains unassessed. Consecutive patients successfully treated with elective TAVR in our center between January 2013 and August 2019 were included. The primary end point was one-year all-cause mortality. TAVR wait times were defined as the interval from referral date for valve replacement to the date of TAVR procedure. A total of 383 patients were included with a mean wait time of 144.2 ± 83.87 days. Death occurred in 55 patients (14.4%) at one year. Increased wait times were independently associated with a relative increase of 1-year mortality by 2% per week after referral (Adjusted Hazard Ratio 1.02 [1.002–1.04]; p = 0.02) for TAVR. Chronic kidney disease, left ventricular ejection fraction ≤ 30%, access site and STS score were other independent correlates of 1-year mortality. Our study shows that wait times are relatively long in routine practice and associated with increased 1-year mortality after successful TAVR. Such findings underscore the need of strategies to minimize delays in access to TAVR.

## Introduction

Transcatheter aortic valve replacement (TAVR) is an effective and increasingly used procedure for patients with severe aortic stenosis (AS). While it represents the sole or preferred strategy in inoperable and high-risk patients^1,2^, it also appears as an alternative to surgical aortic valve replacement for intermediate and low risk patients^3–6^. This paradigm shift in the approach for patients with severe aortic stenosis has led to a rapid increase of TAVR procedures with > 350,000 procedures performed in > 70 countries in the past 15 years^7^. A recent projection estimated that approximately 180,000 patients can be considered potential TAVR candidates annually in the European Union and Northern-America^8^.

This dramatic growth in the demand for TAVR has challenged current capacities which in turn have prolonged wait times^9^. Longer wait-times are associated with increasing mortality and hospitalizations related to heart failure while on the waiting list^9,10^. Moreover, patients referred for TAVR—mostly elderly—could experience a decline in their functional status while waiting^11^. Morbidity associated with wait times may worsen post procedural outcome. The influence of prolonged wait times on late post-TAVR outcomes remains unassessed.

Our study aimed to assess the impact of wait times before TAVR on 1-year mortality in patients successfully treated with TAVR in a real-life cohort.

## Methods

### Study population

All consecutive patients successfully treated with elective TAVR in Caen University Hospital between January 2013 and August 2019 and enrolled in the nationwide FRANCE-TAVI or FRANCE-2 registries^12^, were included in our analysis. Urgent TAVR in patients who required a TAVR procedure during a concurrent hospitalization for a cardiovascular event^13^ were excluded. The registry was approved by the Institutional Review Board of the French Ministry of Higher Education and Research and by the National Commission for Data Protection and Liberties. Patients provided written informed consent before inclusion. All methods were performed in accordance with the relevant guidelines and regulations. The decision to perform TAVR was made by the local heart team after careful individualized evaluation as recommended^14^. All patients underwent a comprehensive transthoracic echocardiography (iE33, Philips, Amsterdam, Netherlands) before TAVR. The severity of aortic stenosis and left ventricular ejection fraction were evaluated according to guidelines^15^. Pre-procedural aortic annular sizing and vascular access assessment were performed using multidetector computed tomography (MDCT). Patients received dual antiplatelet therapy with aspirin and/or clopidogrel, or oral anticoagulation as clinically indicated. Wait times were defined as the interval from referral date for valve replacement to the date of the TAVR.

### Clinical endpoints

The primary end point was all-cause mortality at 1 year. Other outcomes of interest were: rehospitalization for a cardiac event (heart failure, arrhythmia and acute coronary syndrome), computed tomography-scan or magnetic resonance imaging proven stroke or transient ischemic attack, new cardiac pacemaker, acute kidney injury (increase in serum creatinine of 50% or increase of ≥ 0.3 mg/dL compared to baseline) and bleeding defined according to the Valve Academic Research Consortium 2 criteria^16^ at 1-year follow-up.

### Statistical analysis

Patients were separated into two groups defined by their status dead or alive at 1 year follow-up. Continuous variables were expressed as mean ± standard deviation and categorical variables were expressed as numbers of patients and percentages. Baseline characteristics were compared between groups using Student’s t and the Chi^2^ tests where adequate.

The relationship between wait time and 1-year mortality, and the calculation of the Hazard Ratio (HR) and its 95% Confidence Interval (CI), was estimated using Cox regression models, unadjusted and adjusted on pre-specified variables (age, sex, body mass index (BMI), Society for Thoracic Surgeons (STS) score, medical history of diabetes, chronic kidney disease (CKD) and severe left ventricular systolic dysfunction defined as left ventricular ejection fraction, LVEF ≤ 30%). Sensitivity analyses were performed: one analysis (I) adjusted on all variables differently distributed between the two groups (Table 1) and considering wait time as a categorical variable (≤ or > 12 weeks^17^); another analysis adjusted on calendar year periods added to the adjusted model. Additional analysis including only trans-femoral TAVR was added and presented in “Supplementary appendix”. The relationship between wait time and secondary outcomes was assessed using logistic regression models for categorical and continuous variables respectively. A p-value of < 0.05 was considered statistically significant. SAS 9.4 (SAS Institute Inc., Cary. NC. USA) was used for statistical analysis.

**Table 1:** Baseline and procedural characteristics of the study population.

Baseline characteristics	All (N = 383)	Alive (N = 328)	Dead (N = 55)	p
Age (years)	82.9 ± 7.2	83 ± 7.2	82.3 ± 7.6	0.43
Women	192 (50.1%)	168 (51.24%)	24 (43.7%)	0.36
Body mass index (kg/m^2^)	27.3 ± 5.9	27.2 ± 5.5	28 ± 9	0.51
Diabetes mellitus	114 (29.6%)	95 (28.9%)	19 (34.6%)	0.44
Systemic hypertension	232 (60.3%)	198 (60.4%)	34 (61.9%)	0.71
**History of**				
Peripheral artery disease	59 (15.4%)	46 (14%)	13 (23.7%)	0.11
Chronic kidney disease				
All	177 (46%)	140 (42.7%)	37 (67.3%)	0.02
Mild (eGFR 30–59 ml/min)	152 (39.5%)	121 (36.9%)	31 (56.4%)	< 0.01
Severe (eGFR < 30 mL/min)	20 (5.2%)	14 (4.3%)	6 (10.9%)	0.02
Chronic lung disease	74 (19.2%)	62 (18.9%)	12 (21.9%)	0.77
Long term oxygen therapy	6 (1.6%)	4 (1.2%)	2 (3.6%)	0.15
CABG	47 (12.2%)	42 (12.8%)	5 (9.1%)	0.37
SAVR	17 (4.4%)	16 (4.9%)	1 (1.8%)	0.31
PCI	149 (38.7%)	122 (37.2%)	27 (49.1%)	0.15
Stroke	56 (14.6%)	47 (14.3%)	9 (16.4%)	0.77
Pace maker	35 (9.1%)	27 (8.2%)	8 (14.6%)	0.26
Atrial fibrillation or flutter	163 (42.4%)	136 (41.5%)	27 (49.1%)	0.55
Chronic anticoagulation	166 (43.2%)	139 (42.4%)	27 (49.1%)	0.53
NYHA				
Class I	9 (2.34%)	9 (2.8%)	0 (0%)	0.08
Class II	137 (35.6%)	123 (37.5%)	14 (25.5%)	0.04
Class III	197 (51.2%)	163 (49.7%)	34 (61.9%)	0.02
Class IV	40 (10.4%)	33 (10.1)	7 (12.7%)	0.18
STS score	12.3 ± 9.4	11.8 ± 8.4	14.7 ± 13.5	0.03
< 4	49 (12.7%)	47 (14.3%)	2 (3.6%)	0.04
4–8	121 (31.5%)	102 (31.1%)	19 (34.6%)	0.26
> 8–5	121 (31.5%)	101 (30.8%)	14 (25.5%)	0.03
> 15	92 (23.9%)	72 (21.9%)	20 (36.4%)	< 0.01
Poor mobility	31 (8.1%)	28 (8.5%)	3 (5.5%)	0.40
Frailty	116 (30.2%)	95 (28.9%)	21 (38.2%)	0.20
Wait time (days)	144.2 ± 83.87	140.2 ± 77.5	168 ± 113	0.01
**Baseline echocardiography**				
LVEF (%)	57.6 ± 11.9	57.9 ± 11.8	56.2 ± 12.6	0.33
LVEF ≤ 30%	8 (2.1%)	5 (1.5%)	3 (5.5%)	< 0.01
Aortic valve area (cm^2^)	0.85 ± 0.2	0.83 ± 0.23	0.92 ± 0.3	0.57
Aortic valve gradient (mmHg)	46.7 ± 13.8	46.9 ± 13.5	45.7 ± 15.1	0.65
Right ventricular failure	35 (9.1%)	26 (7.9%)	9 (16.4%)	0.03
PASP (mmHg)	41.9 ± 15.4	41.5 ± 15.3	44 ± 16.3	0.25
**TAVR**				
Self-expanding valve	210 (54.6%)	179 (54.6%)	31 (56.4%)	0.37
Balloon-expandable valve	173 (45%)	149 (45.4%)	24 (43.7%)	0.44
Access site				
Femoral	346 (90%)	304 (92.3%)	42 (76.4%)	< 0.01
Sub-Clavian	5 (1.3%)	4 (1.2%)	1 (1.8%)	0.70
Trans-Aortic	13 (3.4%)	11 (3.4%)	2 (3.6%)	0.8
Apical	19 (4.9%)	9 (2.7%)	10 (18.2%)	< 0.01

*CABG* coronary artery bypass graft, *eGFR* estimated glomerular filtration rate, *LVEF* left ventricular ejection fraction, *NYHA* New York Heart Association, *PASP* Pulmonary Artery Systolic Pressure, *PCI* percutaneous coronary intervention, *SAVR* surgical aortic valve replacement, *STS* Society for thoracic surgeons, *TAVR* Transcatheter Aortic Valve Replacement.

## Results

After excluding 35 urgent TAVR, a total of 383 patients were included in our study. Death occurred in 21 (5.5%) patients and 55 (14.4%) patients at 30 days and 1 year, respectively. Baseline demographic and procedural characteristics of the population are detailed in Table 1. The mean wait time was 144.2 ± 83.87 days. Patients who died had higher STS scores (14.7 ± 13.5 vs 11.8 ± 8.4; p = 0.03), more often CKD (67.3% vs 42.7%; p = 0.02), LVEF ≤ 30% (5.5% vs 1.5%; p < 0.01), right ventricular failure (16.4% vs 7.9%; p = 0.03), less trans-femoral approaches (76.4% vs 92.3%; p < 0.01) and longer wait times (168 ± 113 vs 140.2 ± 77.5 days; p = 0.01) compared to those alive at 1 year.

### Correlates of 1 year-mortality

In the unadjusted analysis, wait times, CKD, LVEF ≤ 30% and RV failure were associated with increased, while femoral approach was associated with decreased mortality rates at 1 year (Table 2). In the adjusted analysis, wait times (HR 1.02 [1.002–1.04]; p = 0.02), STS score (HR 1.02 [1.001–1.05]; p = 0.03), CKD (HR 3.51 [1.87–6.3]; p < 0.01) and LVEF ≤ 30% (HR 10.05 [3.8–37]; p < 0.01) remained associated with higher and femoral approach with lower mortality rates at 1 year (HR 0.41 [0.2–0.79]; p < 0.01). Sensitivity analyses confirmed significant associations for wait times, CKD and LVEF ≤ 30%. These results were confirmed when analysis was restricted to trans-femoral TAVR (“Supplementary appendix”).

**Table 2:** Association between variables and 1-year mortality.

	Unadjusted analysis		Adjusted analysis		Sensitivity analysis I		Sensitivity analysis II	
	HR [CI 95%]	p	HR [CI 95%]	p	HR [CI 95%]	p	HR [CI 95%]	p
Wait time^a^	1.02 [1.003–1.04]	0.02	1.02 [1.002–1.04]	0.02	1.02 [1.001–1.05]	0.04	1.01 [1.001–1.02]	0.03
STS score	1.03 [1–1.05]	0.06	1.02 [1.001–1.05]	0.03	1.02 [0.99–1.05]	0.06	1.02 [0.94–1.06]	0.14
Chronic kidney disease	3.3 [1.72 – 5.9]	< 0.01	3.51 [1.87–6.3]	< 0.01	3.3 [1.66–5.93]	< 0.01	2.8 [1.4–5.7]	< 0.01
LVEF ≤ 30%	4.7 [1.64–16.6]	< 0.01	10.5 [3.8–37]	< 0.01	9.2 [2.1–31]	< 0.01	8.6 [2.1–29]	< 0.01
Right ventricular failure	2.2 [1.03–4]	0.04	1.79 [0.82–3.59]	0.15	1.09 [0.46–2.29]	0.79	1.2 [0.25–2.8]	0.54
Femoral	0.34 [0.17–0.61]	< 0.01	0.41 [0.2–0.79]	< 0.01	0.68 [0.21–2.73]	0.84	0.5 [0.12–2.5]	0.32
Apical	4.9 [2.38–9.41]	< 0.01	4.41 [2.21–9.7]	< 0.01	2.8 [0.6–10.8]	0.31	2.1 [0.4–10.1]	0.27

Only variables significantly different between groups are depicted in the table.

*CI* confidence interval, *HR* hazard ratio, *LVEF* left ventricular ejection fraction, *STS* Society for thoracic surgeons, *TAVR* Transcatheter Aortic Valve Replacement.

^a^Wait time considered as continuous variable (per week) except for sensitivity analysis I where it was ≤ versus > 12 weeks.

Adjusted analysis: Adjustment on age, sex, body mass index, STS score, diabetes, chronic kidney disease and severe left ventricular systolic dysfunction (LVEF ≤ 30%).

Sensitivity analysis I: Adjustment on all variables differently distributed between the 2 groups alive or dead (Table 1) and considering wait time as a categorical variable (≤ or > 12 weeks).

Sensitivity analysis II: Calendar year periods added to the adjusted model.

Apart from 1-year mortality, other outcomes were not significantly associated with wait times (Table 3). Echocardiography at 1 year follow-up is described in the “Supplementary appendix”.

**Table 3:** Association between wait time and outcomes.

Outcomes	All patients (n = 383)	Unadjusted analysis		Adjusted analysis		Sensitivity analysis I		Sensitivity analysis II	
		HR [CI 95%]	p	HR [CI 95%]	p	HR [CI 95%]	p	HR (CI 95%)	p
**All-cause death**									
In hospital	15 (3.9%)	0.97 [0.93–1.01]	0.76	0.98 [0.95–1.03]	0.81	0.98 [0.91–1.07]	0.61	0.99 [0.96–1.03]	0.76
At 30 days	21 (5.5%)	1.02 [0.99–1.05]	0.21	1.19 [0.99–1.06]	0.47	1.02 [0.98–1.14]	0.31	1.01 [0.96–1.13]	0.44
At 1 year	55 (14.4%)	1.02 [1.003–1.04]	0.02	1.02 [1.002–1.04]	0.02	1.02 [1.001–1.05]	0.04	1.01 [1.001–1.02]	0.03
Death from Cardiac causes at 1 year	34 (8.9%)	0.98 [0.96–1.03]	0.89	1.01 [0.99–1.05]	0.61	1 [0.97–1.035]	0.80	1 [0.97–1.04]	0.71

Outcomes at 1 year		Odds Ratio [CI 95%]	p	Odds Ratio [CI 95%]	p	Odds Ratio [CI 95%]	p	Odds Ratio [CI 95%]	p
**Re hospitalization for cardiac event**									
All	52 (13.6%)	1.01 [0.98–1.03]	0.33	1.01 [0.98–1.04]	0.27	1.01 [0.98–1.04]	0.41	1.01 [0.98–1.04]	0.49
Heart failure	37 (9.7%)	1.01 [0.97–1.03]	0.56	1.01 [0.98–1.04]	0.11	1.01 [0.97–1.04]	0.58	1.01 [0.98–1.04]	0.54
Arrhythmia	5 (1.3%)	1.03 [0.88–1.3]	0.71	1.04 [0.88–1.22]	0.89	1.02 [0.88–1.2]	0.79	1.02 [0.88–1.2]	0.90
Myocardial infarction	10 (2.6%)	1.02 [0.95–1.1]	0.44	1.02 [0.96–1.14]	0.71	1.02 [0.96–1.13]	0.46	1.02 [0.96–1.13]	0.63
Stroke or transient ischemic attack	11 (2.9%)	1.01 [0.96–1.06]	0.85	0.99 [0.97–1.02]	0.71	0.99 [0.97–1.02]	0.80	0.99 [0.97–1.02]	0.77
**Bleeding**									
All	74 (19.3%)	0.99 [0.96–1,009]	0.23	0.99 [0.96–1.007]	0.21	0.99 [0.97–1.006]	0.19	0.99 [0.97–1.009]	0.31
Minor bleeding	35 (9.1%)	0.97 [0.96–1.003]	0.11	0.99 [0.95–1.002]	0.07	0.98 [0.95–1.002]	0.08	0.98 [0.95–1.002]	0.23
Major bleeding	15 (3.9%)	0.99 [0.93–1.027]	0.50	0.98 [0.92–1.029]	0.49	0.98 [0.93–1.029]	0.57	0.98 [0.91–1.029]	0.73
Life threatening or disabling bleeding	24 (6.3%)	1.01 [0.99–1.044]	0.51	1.02 [0.99–1.045]	0.50	1.02 [0.997–1.067]	0.31	1.02 [0.998–1.07]	0.42
New pacemaker	56 (14.6%)	0.99 [0.97–1.02]	0.55	0.99 [0.97–1.02]	0.65	0.99 [0.97–1.02]	0.71	0.99 [0.97–1.02]	0.63
Acute kidney injury	27 (7.1%)	0.99 [0.93–1.023]	0.67	0.99 [0.92–1.024]	0.72	0.99 [0.92–1.025]	0.86	0.99 [0.91–1.026]	0.71

*CI* confidence interval, *HR* hazard ratio.

^a^Wait time considered as continuous variable (per week) except for sensitivity analysis I where it was ≤ versus > 12 weeks.

Multivariate adjusted analysis: Adjustment on age, sex, body mass index, STS score, diabetes, chronic kidney disease and severe left ventricular systolic dysfunction (LVEF ≤ 30%).

Sensitivity analysis I: Adjustment on all variables differently distributed between the 2 groups alive or dead (Table 1) and considering wait time as a categorical variable (≤ or > 12 weeks).

Sensitivity analysis II: Adjustment on calendar year periods.

## Discussion

Mortality at 1 year follow-up remains important after TAVR. The wait time before TAVR was relatively important in our cohort and associated with a 2% per week increase of rates of 1-year mortality independent of other correlates of mortality. CKD, LVEF ≤ 30%, access site and STS score were other independent correlates of mortality.

The expansion of TAVR indications has rapidly challenged current capacities leading to a progressive increase in wait times over the last decade^9^. The mean wait time in our study (144 days) appears important. This remains poorly described in the literature with a mean time varying between 89 and 132 days^9,10,13,18^. Such delay in our cohort may be explained by the fact that our center is a tertiary reference center for a mostly rural region.

Previous studies have reported that 2–14% of patients referred for TAVR die while on the waiting list^10,18,19^. Recently, a study focused on the impact of wait times on early post-TAVR outcomes and found a somewhat unexpected higher hazard associated with short wait times^13^. This relationship was entirely mediated by the emergency status with urgent patients having worse outcomes. After adjusting on this point, there was no longer a relationship between wait times and 30-day mortality. Our study confirms the lack of association between wait times and early mortality in elective TAVR patients. However, we found a significant relationship between wait times and 1-year mortality with a relative increase of 2% per week after referral. Sensitivity analyses confirmed this result taking into account other covariables, the possible technical improvements over the study period or the preferential use of transfemoral access. Our data are consistent with a prior study showing dramatically higher 1-year mortality in patients who undergo TAVR after an initial refusal^20^.

Our findings may be explained by a decline of the patients’ functional status while on the waiting list. Decreased cognition, alteration of mobility and renal function are known factors of poor outcomes and recovery after TAVR^21^. Previous studies have reported that frailty scores, which are known to be associated with 1-year mortality^22^, worsened in elderly patients while waiting for TAVR^11^. Additionally, myocardial overload may get worse leading to heart failure and reduced ventricular function which represents a prognostic turning point in AS^23,24^.

Previous studies and our results highlight the need for strategies to minimize delays in access to TAVR and identifying high-risk patients who require a faster processing. Knowing the need of multiple disciplines to evaluate these patients^25^, optimal coordination of care may reduce wait times. The simplification of the procedure performed under local anesthesia which are faster and consume less medical resources, represent a valuable option^26,27^. Individualized risk stratification to consider the urgency of the TAVR is important. The recent position paper of the Canadian Cardiovascular Society recommends performing TAVR within 2 weeks for urgent cases and within 12 weeks for elective cases^17^. Several conditions highlighted in our study and others, such as CKD, STS score, left ventricular systolic dysfunction^18,19,21,28^ may help determining the ideal timing of TAVR.

### Limitations

Given the observational nature of the study, different known or unknown correlates of outcomes may have not been considered in the analyses. The time from referral to TAVR may underestimate the magnitude of effect of longer wait times on outcomes as compared to the time symptom onset (unknown in our cohort). However, our definition of wait times before TAVR remains the most accurately assessable and widely used^10,13^. Finally, our center is the reference center of a semi-rural region and our results may not apply to all regions.

## Conclusion

Patients awaiting TAVR represent a growing population. Our study shows that wait times remain important in daily practice and are associated with a 2% per week increase of 1-year mortality after referral. Our findings underscore the need for physicians and health system administrators to minimize such delays in order to improve prognosis.

## Supplementary Information

Supplementary Tables.
