# Supplementary material for: Impact of wait times on late postprocedural mortality after successful transcatheter aortic valve replacement
Source: Sci Rep. 2022 Apr 8;12:5967. doi: 10.1038/s41598-022-09995-z (PMC8993919; doi:10.1038/s41598-022-09995-z)
Supplement: Supplementary file 1 — Supplementary Tables. [file 41598_2022_9995_MOESM1_ESM.docx]

**Supplementary Appendix**

**Supplementary Table 1:** Association between variables and 1-year mortality in femoral TAVR patients (n=346).

|  | Unadjusted analysis |  | Adjusted analysis |  | Sensitivity analysis I |  |
| --- | --- | --- | --- | --- | --- | --- |
|  | **HR [CI 95%]** | **p** | **HR [CI 95%]** | **p** | **HR [CI 95%]** | **p** |
| Wait time * | 1.02 [1.003-1.05] | 0.02 | 1.02 [1.003-1.05] | 0.01 | 1.02 [1.001-1.05] | 0.04 |
| Chronic kidney disease | 2.8 [1.6 – 5.1] | <0.01 | 3.2 [1.75-6.1] | <0.01 | 3.5 [1.7-5.9] | <0.01 |
| LVEF ≤30% | 5.8 [1.61-17.3] | <0.01 | 10.54 [3.80-36] | <0.01 | 9.5 [2.22-32.3] | <0.01 |
| Right ventricular failure | 2.1 [1.01-4] | 0.03 | 1.78 [0.85-3.6] | 0.13 | 1.04 [0.48-2.27] | 0.93 |
| CI=Confidence Interval; HR= Hazard Ratio; LVEF=Left ventricular ejection fraction; STS=Society for thoracic surgeons; TAVR=Transcatheter Aortic Valve Replacement | | | | | | |

Only variables significantly different between groups are depicted in the table

* Wait time considered as continuous variable (per week) except for sensitivity analysis I where it was ≤ versus > 12weeks

**Adjusted analysis:** Adjustment on age, sex, body mass index, STS score, diabetes, chronic kidney disease and severe left ventricular systolic dysfunction (LVEF≤30%).

**Sensitivity analysis I:** Adjustment on all variables differently distributed between the 2 groups alive or dead (table 1) and considering wait time as a categorical variable (≤ or > 12 weeks).

**Supplementary Table 2:** Association between wait time and outcomes in femoral TAVR patients.

| Outcomes | All patients  (n=346) | Unadjusted analysis |  | Adjusted analysis |  | Sensitivity analysis I |  |
| --- | --- | --- | --- | --- | --- | --- | --- |
|  |  | **HR [CI 95%]** | **p** | **HR [CI 95%]** | **p** | **HR [CI 95%]** | **p** |
| All-cause Death |  |  |  |  |  |  |  |
| In hospital | 12 (3.5%) | 0.99 [0.95-1.03] | 0.88 | 0.99 [0.96-1.04] | 0.75 | 0.97 [0.90-1.06] | 0.65 |
| At 30 days | 16 (4.7%) | 1.02 [0.97-1.06] | 0.24 | 1.02 [0.97-1.07] | 0.62 | 1.02 [0.96-1.11] | 0.31 |
| At 1 year | 42 (12.3%) | 1.016 [1.003-1.05] | 0.02 | 1.02 [1.003-1.05] | 0.01 | 1.02 [1.001-1.05] | 0.04 |
| Death from Cardiac causes at 1 year | 28 (8.2%) | 0.94 [0.94-1.03] | 0.93 | 1 [0.95-1.05] | 0.79 | 1 [0.95-1.037] | 0.96 |
| Outcomes at 1 year |  | **Odds Ratio [CI 95%]** | **p** | **Odds Ratio [CI 95%]** | **p** | **Odds Ratio [CI 95%]** | **p** |
| Re hospitalization for cardiac event |  |  |  |  |  |  |  |
| All | 48 (14%) | 1.01 [0.98-1.03] | 0.41 | 1.01 [0.96-1.04] | 0.27 | 1.01 [0.97-1.04] | 0.44 |
| Heart failure | 34 (9.9%) | 1.01 [0.98-1.03] | 0.60 | 1.01 [0.98-1.04] | 0.22 | 1.01 [0.96-1.04] | 0.69 |
| Arrhythmia | 5 (1.5%) | 1.04 [0.90-1.23] | 0.44 | 1.04 [0.89-1.21] | 0.77 | 1.02 [0.87-1.2] | 0.69 |
| Myocardial infarction | 9 (2.6%) | 1.04 [0.95-1.09] | 0.39 | 1.03 [0.96-1.1] | 0.65 | 1.03 [0.96-1.1] | 0.51 |
| Stroke or transient ischemic attack | 11 (3.2%) | 1.01 [0.96-1.06] | 0.94 | 0.99 [0.97-1.02] | 0.66 | 0.99 [0.97-1.02] | 0.70 |
| Bleeding |  |  |  |  |  |  |  |
| All | 67 (19.6%) | 0.98 [0.95-1,01] | 0.22 | 0.99 [0.97-1.01] | 0.21 | 0.99 [0.97-1.006] | 0.30 |
| Minor bleeding | 31 (9.1%) | 0.98 [0.95-1.004] | 0.17 | 0.99 [0.95-1.002] | 0.07 | 0.98 [0.95-1.002] | 0.09 |
| Major bleeding | 15 (4.4%) | 0.99 [0.95-1.03] | 0.50 | 0.98 [0.95-1.03] | 0.43 | 0.98 [0.95-1.03] | 0.53 |
| Life threatening or disabling bleeding | 21 (6.1%) | 1.01 [0.98-1.05] | 0.44 | 1.02 [0.98-1.06] | 0.49 | 1.02 [0.98-1.07] | 0.34 |
| New pacemaker | 53 (15.5%) | 0.99 [0.97-1.02] | 0.61 | 0.99 [0.97-1.02] | 0.70 | 0.99 [0.97-1.02] | 0.69 |
| Acute kidney injury | 21 (6.1%) | 0.99 [0.96-1.02] | 0.69 | 0.99 [0.96-1.02] | 0.71 | 0.99 [0.97-1.03] | 0.91 |
| CI=Confidence Interval; HR= Hazard Ratio; TAVR=Transcatheter Aortic Valve Replacement | | | | | | | |

* Wait time considered as continuous variable (per week) except for sensitivity analysis I where it was ≤ versus > 12weeks

**Adjusted analysis:** Adjustment on age, sex, body mass index, STS score, diabetes, chronic kidney disease and severe left ventricular systolic dysfunction (LVEF≤30%).

**Sensitivity analysis I:** Adjustment on all variables differently distributed between the 2 groups alive or dead (table 1) and considering wait time as a categorical variable (≤ or > 12 weeks).

**Supplementary Table 3:** Echocardiography at 1 year follow-up after TAVR

| Echocardiography at 1 year | N=328 |
| --- | --- |
| LVEF (%) | 57.6 ± 11 |
| LVEF ≤30% | 3 (0,8) |
| Aortic Valve Area (cm2) | 2,24 ± 4.9 |
| Aortic Valve Gradient (mmHg) | 10.9 ± 6.24 |
| Right ventricular failure | 30 (7.8%) |
| PASP (mmHg) | 41.9 ± 13.4 |
| LVEF=Left ventricular ejection fraction; PASP: Pulmonary Artery Systolic Pressure; TAVR=Transcatheter Aortic Valve Replacement | |
